# Primary Sjögren’s Syndrome Revealed During the Evaluation of Paroxysmal Tonic Spasms Leading to the Diagnosis of Seronegative Neuromyelitis Optica Spectrum Disorder: A Case Report

**DOI:** 10.7759/cureus.109666

**Published:** 2026-05-26

**Authors:** Hajar Hanane, Zakaria Salimi, AbdelHamid Naitlhou, Moulay Berkchi Jihad, Lahsen Achemlal

**Affiliations:** 1 Rheumatology, Faculty of Medicine, Mohammed VI University of Health Sciences (UM6SS), Casablanca, MAR; 2 Neurology, Mohammed VI University of Health Sciences (UM6SS), Casablanca, MAR; 3 Internal Medicine, Mohammed VI University of Health Sciences (UM6SS), Casablanca, MAR; 4 Rheumatology, Mohammed VI University of Health Sciences (UM6SS), Rabat, MAR

**Keywords:** autoimmune diseases, longitudinally extensive transverse myelitis (letm), paroxysmal tonic spasms, primary sjögren’s syndrome, seronegative nmosd

## Abstract

Neuromyelitis optica spectrum disorder (NMOSD) may coexist with other autoimmune diseases, including primary Sjögren’s syndrome. We report a 52-year-old female presenting with sicca symptoms leading to the diagnosis of primary Sjögren’s syndrome based on a positive Schirmer’s test, grade III sialadenitis, and anti-SSB antibodies. Further evaluation revealed a history of optic neuritis and paroxysmal tonic spasms involving the limbs, associated with intractable hiccups and acute myelopathy. Magnetic resonance imaging showed brain and spinal lesions suggestive of NMOSD, with negative aquaporin-4 (AQP4) and anti-myelin oligodendrocyte glycoprotein (MOG) antibodies. A diagnosis of seronegative NMOSD associated with primary Sjögren’s syndrome was retained. Immunosuppressive therapy led to clinical improvement. This case highlights that paroxysmal tonic spasms may reveal NMOSD in patients with Sjögren’s syndrome.

## Introduction

Primary Sjögren’s syndrome is a systemic autoimmune disease characterized by lymphocytic infiltration of the exocrine glands, typically leading to sicca symptoms. However, it may also present with systemic and neurological manifestations, including severe central nervous system involvement [1].

Neuromyelitis optica spectrum disorder (NMOSD) is an inflammatory, autoimmune demyelinating disorder of the central nervous system that predominantly targets the optic nerves and spinal cord. While it is most commonly driven by pathogenic anti-aquaporin-4 (AQP4) antibodies, seronegative forms are well recognized [2]. NMOSD can occur in isolation or concurrently with other autoimmune diseases, particularly Sjögren’s syndrome, suggesting shared immunopathogenic pathways [2].

The coexistence of these two conditions complicates the diagnostic process, especially when complex neurological manifestations precede or obscure classical sicca symptoms. Paroxysmal tonic spasms (PTS), i.e., brief, involuntary, often painful dystonic episodes, frequently stem from spinal cord lesions and can represent an early clinical warning sign of NMOSD [3]. Early recognition of this overlap syndrome is vital to initiating targeted immunosuppressive therapy and mitigating irreversible neurological disability.

## Case presentation

A 52-year-old female with a medical history of hypertension and type 2 diabetes mellitus was referred to our department for the evaluation of chronic sicca symptoms, including progressive xerophthalmia and xerostomia over several months. She also reported systemic symptoms of fatigue and intermittent arthralgia without objective joint swelling.

Initial clinical evaluation confirmed marked ocular dryness with a positive Schirmer’s test (<5 mm/5 minutes), and an oral examination revealed significantly decreased salivary flow. An autoimmune workup demonstrated high-titer antinuclear antibodies (ANA) with a speckled pattern (1:640) and positive anti-SSB antibodies, whereas anti-SSA antibodies were negative. A labial salivary gland biopsy revealed grade III focal lymphocytic sialadenitis according to the Chisholm-Mason classification [4]. Collectively, these findings fulfilled the 2016 American College of Rheumatology (ACR)/European Alliance of Associations for Rheumatology (EULAR) classification criteria for primary Sjögren’s syndrome [5].

A detailed retrospective history taking and neurological anamnesis revealed an overlapping, five-year timeline of neurological symptoms. Her neurological history began five years prior with an episode of severe right retrobulbar optic neuritis, which had resulted in persistent visual impairment. This initial event was followed by recurrent PTS involving both the lower and upper limbs, described as brief, painful, involuntary dystonic contractions triggered by movement or tactile stimuli. She also reported a historical episode of intractable hiccups and vomiting lasting for over a week, which had previously been managed symptomatically without a definitive diagnosis.

The clinical picture culminated in an episode of acute severe myelopathy characterized by rapid deterioration of motor and sensory function, with the Expanded Disability Status Scale (EDSS) score reaching 8.0 at nadir within one week [6]. During this acute longitudinally extensive transverse myelitis (LETM) episode, a comprehensive neurological examination was conducted.

Neuro-ophthalmologic findings were as follows: visual acuity was reduced to finger counting at 1 meter in the right eye, accompanied by a relative afferent pupillary defect (RAPD) and temporal disc pallor on fundoscopy. Visual acuity in the left eye was 20/20 with normal fundoscopic findings.

Apart from the right optic nerve pathology (cranial nerve II), cranial nerves III through XII were completely intact.

Motor examination revealed spastic quadriparesis, which was more pronounced in the lower extremities. Motor strength, graded via the Medical Research Council (MRC) scale [7], was 3/5 in the upper limbs proximally and distally, and 1/5 in the lower limbs bilaterally. Marked spasticity (Modified Ashworth Scale grade 3 [8]) was noted in both lower extremities.

Reflexes and pyramidal signs were as follows: deep tendon reflexes were diffusely hyperactive (3+) in the upper limbs and prominent (4+) at the knees and ankles, with sustained bilateral ankle clonus. Pathological pyramidal signs, including positive bilateral Babinski and Hoffmann signs, were present.

Sensory system examination revealed the following: a distinct, dense sensory level to pain, temperature, and light touch was mapped to the T4 dermatome. Proprioception and vibration sense were severely impaired in both lower extremities.

Gait and autonomic function examination revealed the following: the patient was entirely non-ambulatory, bedridden due to the severe paretic deficit, and exhibited autonomic dysfunction presenting as urinary urgency and retention requiring intermittent catheterization.

Urgent spinal cord and brain magnetic resonance imaging (MRI) was performed. Spinal MRI demonstrated a longitudinally extensive intramedullary T2-hyperintense lesion extending continuously over more than three vertebral segments involving the cervicothoracic spinal cord, demonstrating "bright spotty lesions" on short tau inversion recovery (STIR) sequences and patchy post-gadolinium enhancement (Figure 1). Brain MRI revealed T2/fluid-attenuated inversion recovery (FLAIR) hyperintense lesions localized to the brainstem, specifically involving the area postrema (Figure 2).

**Figure 1:**
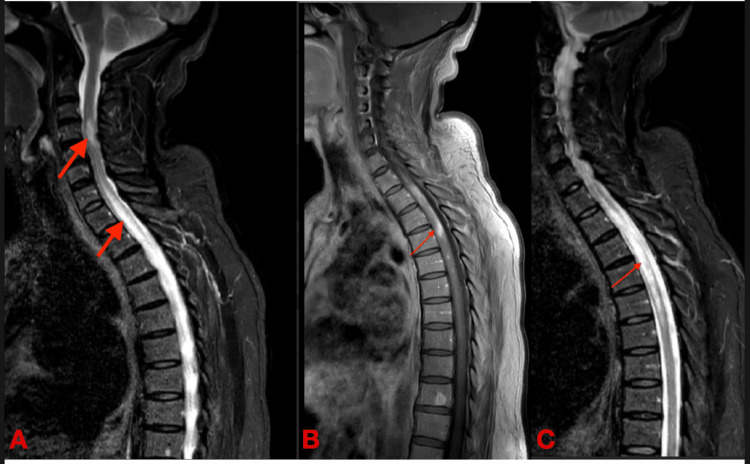
Spinal cord MRI findings in NMOSD. (A) Sagittal T2-weighted image showing a longitudinally extensive intramedullary hyperintensity involving the cervicothoracic spinal cord (red arrows). (B) Sagittal STIR sequence demonstrating marked thoracic cord hyperintensity with characteristic bright spotty lesions (red arrows). (C) Sagittal T1-weighted image after gadolinium administration showing patchy intramedullary enhancement (red arrows). MRI: magnetic resonance imaging; NMOSD: neuromyelitis optica spectrum disorder; STIR: short tau inversion recovery.

**Figure 2:**
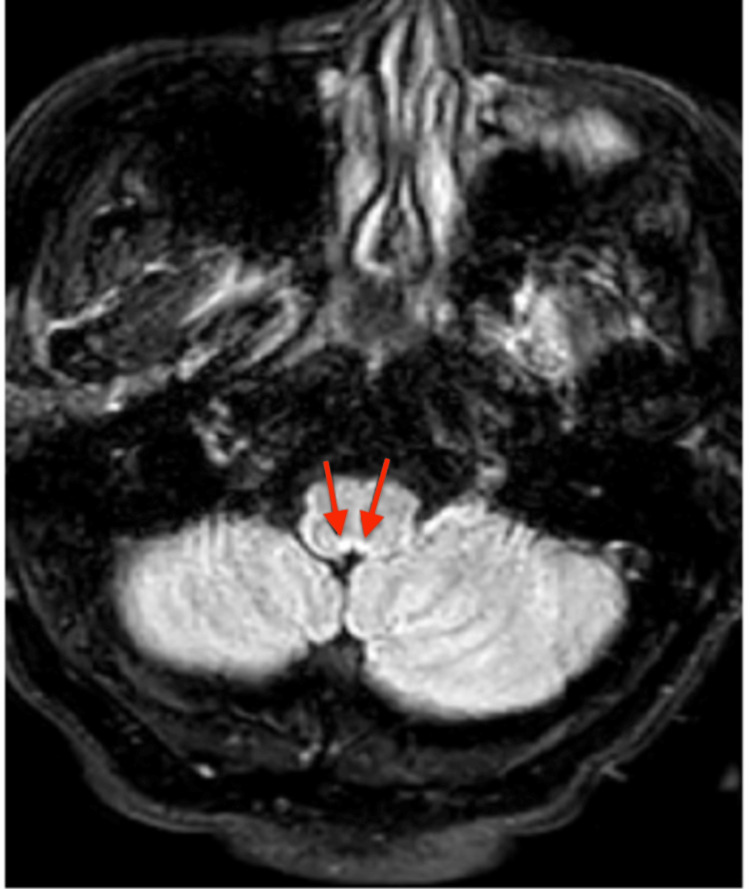
Brain MRI demonstrating encephalic lesions involving the area postrema in NMOSD (red arrows). MRI: magnetic resonance imaging; NMOSD: neuromyelitis optica spectrum disorder

Serum cell-based assays for anti-aquaporin-4 (AQP4-IgG) and anti-myelin oligodendrocyte glycoprotein (MOG) antibodies were repeatedly negative. Cerebrospinal fluid (CSF) analysis revealed mild lymphocytic pleocytosis (18 cells/μL) and elevated protein (0.65 g/L) without oligoclonal bands (Table 1). Based on the 2015 International Consensus Diagnostic Criteria, a diagnosis of seronegative NMOSD coexisting with primary Sjögren's syndrome was established [2].

**Table 1:** Laboratory and diagnostic findings with reference ranges. ESR: erythrocyte sedimentation rate; CRP: C-reactive protein; ANA: antinuclear antibodies; Anti-SSA (Ro): anti-Sjögren syndrome-related antigen A (Ro); Anti-SSB (La): anti-Sjögren syndrome-related antigen B (La); Anti-AQP4: anti-aquaporin-4 antibody; Anti-MOG: anti-myelin oligodendrocyte glycoprotein antibody; SGB: salivary gland biopsy; LSGB: labial salivary gland biopsy; NMOSD: neuromyelitis optica spectrum disorder; MOGAD: myelin oligodendrocyte glycoprotein antibody-associated disease.

Parameter	Result	Reference range	Interpretation
Hemoglobin	12.8 g/dL	12-16 g/dL	Normal
White blood cells	6.4 ×10⁹/L	4-10 ×10⁹/L	Normal
Platelets	240 ×10⁹/L	150-400 ×10⁹/L	Normal
ESR	45 mm/h	<20 mm/h	Elevated
CRP	12 mg/L	<5 mg/L	Mildly elevated
ANA	Positive 1:640 (speckled)	Negative	Positive autoimmune profile
Anti-SSA (Ro)	Negative	Negative	Negative
Anti-SSB (La)	Positive	Negative	Consistent with Sjögren’s syndrome
AQP4-IgG	Negative	Negative	Seronegative NMOSD
Anti-MOG antibodies	Negative	Negative	MOGAD excluded
Schirmer test	<5 mm/5 min	>10 mm/5 min	Severe ocular dryness
Labial salivary gland biopsy	Grade III sialadenitis	Chisholm-Mason grade 0-IV	Compatible with primary Sjögren’s syndrome

The patient was treated aggressively during the acute phase with high-dose intravenous methylprednisolone (1 g/day for seven consecutive days), followed by an oral prednisone taper. For long-term maintenance and relapse prevention, immunosuppressive therapy with rituximab (1 g intravenously, two doses two weeks apart, followed by maintenance every six months) was initiated. This therapeutic regime resulted in significant clinical recovery: her motor strength improved to 4+/5 in the upper limbs and 4/5 in the lower limbs, she regained the ability to ambulate with assistance, and there was a marked reduction in both the intensity and frequency of her PTS.

## Discussion

Primary Sjögren’s syndrome is a systemic autoimmune disease classically characterized by lymphocytic infiltration of the exocrine glands. However, extra-glandular manifestations are common and frequently target both the peripheral and central nervous systems (CNS). Notably, CNS involvement can precede classical sicca symptoms by years, making the initial diagnostic workup particularly challenging [1,9].

NMOSD is a rare autoimmune disease of the CNS marked by immune-driven inflammation and damage targeting astrocytes. While predominantly driven by pathogenic anti-AQP4 antibodies, seronegative cases represent a well-recognized subset that mirrors the classical clinical phenotype of recurrent optic neuritis and LETM [10]. The 2015 International Consensus Diagnostic Criteria explicitly accommodate the diagnosis of seronegative NMOSD, provided that the core clinical characteristics, such as LETM, optic neuritis, or area postrema syndrome, are accompanied by characteristic MRI lesions and the rigorous exclusion of alternative diagnoses [2].

The coexistence of NMOSD and primary Sjögren’s syndrome is an increasingly reported phenomenon, pointing toward shared immunopathogenic mechanisms rooted in B-cell dysregulation and systemic autoantibody production [11]. In clinical practice, Sjögren’s syndrome may precede the neurological onset or, as observed in our patient, be diagnosed concurrently during the evaluation of an acute neuro-inflammatory event.

Paroxysmal tonic spasms are brief, stereotypic, involuntary dystonic episodes that stem from axonal hyperexcitability within damaged spinal cord tracts [3]. They are highly characteristic of NMOSD and serve as an early clinical marker of active myelitis or an impending disease relapse [12]. Recognizing PTS is clinically vital because these spasms frequently precede overt, irreversible transverse myelitis. Similarly, severe or recurrent optic neuritis serves as a hallmark feature of NMOSD that frequently predates extensive spinal cord involvement by several years [13].

Neuroimaging remains indispensable for diagnostic differentiation. An MRI finding of LETM spanning three or more contiguous vertebral segments strongly differentiates NMOSD from multiple sclerosis and other inflammatory myelopathies [12,14]. Furthermore, the presence of specific T2/FLAIR hyperintensities within the floor of the fourth ventricle explains the patient’s history of intractable hiccups and vomiting. This area postrema syndrome serves as a highly specific, anatomically localized diagnostic criterion for NMOSD [12].

Navigating seronegative NMOSD within an autoimmune overlap syndrome presents a unique therapeutic challenge, demanding the early integration of clinical, radiological, and serological parameters. Prompt intervention is critical to mitigate the risk of accumulating irreversible neurological deficits. Long-term maintenance therapy in NMOSD fundamentally aims to suppress relapses and preserve the functional baseline.

While foundational steroid-sparing immunosuppressive agents include rituximab, azathioprine, and mycophenolate mofetil, the therapeutic landscape has expanded to include newer, targeted biologic therapies. For instance, satralizumab, an interleukin-6 (IL-6) receptor antagonist, has demonstrated significant efficacy in preventing relapses, offering a tailored option for patients with highly active or recurrent disease [15].

Ultimately, the sequential evolution of optic neuritis, paroxysmal tonic spasms, extensive transverse myelopathy, and subsequent sicca symptoms in our patient underscores the vital need for a low threshold of suspicion for systemic autoimmune overlap syndromes. Early, aggressive immunosuppressive therapy remains the definitive cornerstone of management for both NMOSD and Sjögren’s syndrome to halt disability progression.

## Conclusions

The coexistence of primary Sjögren’s syndrome and NMOSD highlights the importance of considering CNS involvement in patients with systemic autoimmune diseases presenting with neurological symptoms. Paroxysmal tonic spasms may represent an early and underrecognized clinical marker of disease activity or relapse in NMOSD. Early identification of this association is crucial to avoid diagnostic delay and to initiate prompt immunosuppressive therapy, which may improve neurological prognosis and limit long-term disability.
